# A note on generalized Genome Scan Meta-Analysis statistics

**DOI:** 10.1186/1471-2105-6-32

**Published:** 2005-02-17

**Authors:** James A Koziol, Anne C Feng

**Affiliations:** 1 Department of Molecular and Experimental Medicine, The Scripps Research Institute, 10550 North Torrey Pines Road, MEM216, La Jolla, CA 92037, USA

## Abstract

**Background:**

Wise et al. introduced a rank-based statistical technique for meta-analysis of genome scans, the Genome Scan Meta-Analysis (GSMA) method. Levinson et al. recently described two generalizations of the GSMA statistic: (i) a weighted version of the GSMA statistic, so that different studies could be ascribed different weights for analysis; and (ii) an order statistic approach, reflecting the fact that a GSMA statistic can be computed for each chromosomal region or bin width across the various genome scan studies.

**Results:**

We provide an Edgeworth approximation to the null distribution of the weighted GSMA statistic, and, we examine the limiting distribution of the GSMA statistics under the order statistic formulation, and quantify the relevance of the pairwise correlations of the GSMA statistics across different bins on this limiting distribution. We also remark on aggregate criteria and multiple testing for determining significance of GSMA results.

**Conclusion:**

Theoretical considerations detailed herein can lead to clarification and simplification of testing criteria for generalizations of the GSMA statistic.

## Background

Wise, Lanchbury and Lewis [1] introduced a rank-based statistical technique for meta-analysis of genome scans, the Genome Scan Meta-Analysis (GSMA) method, and derived its exact null distribution using a clever inclusion/exclusion argument. Koziol and Feng [2] provided an alternative derivation of the null distribution of the GSMA statistic via a combinatoric approach involving probability generating functions, and suggested an Edgeworth series approximation to its exact null distribution that improves upon the Wise [1] normal approximation.

Levinson [3] described two generalizations to the GSMA statistic: (i) a weighted version of the GSMA statistic, so that different studies could be ascribed different weights for analysis; and (ii) an order statistic approach, reflecting the fact that a GSMA statistic can be computed for each chromosomal region or bin across the various genome scan studies. Wise [1] had suggested that each chromosomal region (bin) be about 30 cM, leading to a total of about *n *= 120 bins spanning the entire genome, and correspondingly 120 GSMA statistics. Wise [1] and Koziol and Feng [2] had investigated the marginal distribution of any of these (exchangeable) GSMA statistics, whereas under the order statistic formulation of Levinson [3], the joint distribution of the entire set of GSMA statistics is taken into account. In this note, we consider both generalizations in turn. In particular, (i) we provide an Edgeworth approximation to the null distribution of the weighted GSMA statistic, analogous to that in Koziol and Feng [2]; and (ii) we examine the limiting distribution of the GSMA statistics under the order statistic formulation, and quantify the relevance of the pairwise correlations of the GSMA statistics across different bins on this limiting distribution. We conclude with remarks concerning the Levinson [3] aggregate criteria and multiple testing for determining significance of GSMA results.

## Results

### The GSMA statistics

We first introduce some notation. Let *X*_*ij*_, *i *= 1, ..., *m*, *j *= 1, ..., *n*, denote the rank of any particular linkage test statistic (e.g., LOD score) in the *j*^*th *^chromosomal region (bin) from the *i*^*th *^study, with each study being ranked separately. Levinson [3] rank the bins from 1 = "best" to *n *= "worst" on the basis of, say, maximum LOD score or lowest *p *value observed within each bin, but the reverse ranking from 1 = "worst" to *n *= "best" is also feasible. In practice, *m *can be as few as 4 (e.g., [4,5]); and, following Wise [1], *n *is generally about 120. The GSMA statistics are then *S*_1_, ..., *S*_*n*_, where 
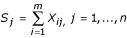
. The exact (marginal) null distribution of each *S*_*j *_was derived in Wise [1]; in the notation of Levinson [3], *P*_*AvgRnk*_, the "pointwise probability" of any *S*_*j*_, is obtained from its marginal null distribution. The normal approximation to the exact distribution of the *S*_*j *_is straightforward: the *S*_*j *_are identically distributed, and each *S*_*j *_has an approximate normal distribution with mean 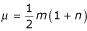 and variance 
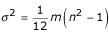
 under the null hypothesis that ranks are randomly assigned within each study. Koziol and Feng [2] provided an Edgeworth correction to this approximation, and recommended that the correction be used, at least for *m *≤ 12.

### The weighted GSMA statistic

Levinson [3] proposed a weighted version of the GSMA statistic, namely, 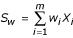, with the weight *w*_*i *_ascribed to the *i*^*th *^study reflecting the relative linkage information from that study. (We are temporarily omitting the *j *subscript for clarity.) The normal approximation to the marginal null distribution of *S*_*w *_is straightforward, and depends on the two parameters 
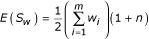
 and 
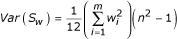
. The combinatorial argument utilized by Koziol and Feng [2] to derive the exact distribution of the unweighted GSMA statistic (which relies on probability generating functions) is generally no longer applicable in the weighted setting. Nevertheless, as in Koziol and Feng [2], we here provide an Edgeworth correction that may be applied to the weighted GSMA statistic. To this end, we equivalently consider the linear transform 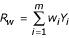, where 
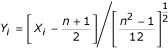
. We then have *E*[*R*_*w*_] = 0, 
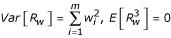
, and


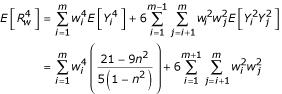


(We have used Koziol and Feng [2], eqn. 11, for 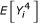.)

The Edgeworth Type A series approximation to the density of 
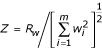
 up to 4^th ^order terms, is *f *(*z*) = *φ *(*z*)[1 + *c*_4_*H*_4 _(*z*)], where *φ*(·) is the standard normal density function, 
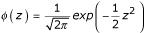
, *H*_4 _(·) is the 4^th ^degree Hermite-Chebyshev polynomial, *H*_4 _(*z*) = *z*^4 ^- 6*z*^2 ^+ 3, and the constant *c*_4 _given by 
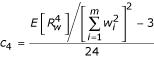
 (Stuart and Ord [6], eqn. 6.42). Furthermore, the cumulative distribution function of the Edgeworth series is given simply by


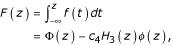


where Φ(·) denotes the cumulative distribution function of the standard normal distribution, *c*_4 _and *φ*(·) are as above, and *H*_3_(·) is the 3^rd ^degree Hermite-Chebyshev polynomial, *H*_3 _(*z*) = *z*^3 ^- 3*z *(Stuart and Ord [6], eqn. 6.43).

In practice, we would expect the Edgeworth series approximation to provide an adequate representation of the exact distribution of the weighted GSMA statistic, in a manner analogous to the unweighted case [2]. Here, we briefly investigate the adequacy of the Edgeworth approximation, using an example from Lewis et al. [7]. They had applied the GSMA methodology to data from *m *= 20 schizophrenia genome scans, and found strong evidence for linkage on chromosome 2q, as well as suggestive evidence for linkage at several other chromosomal locations. The rank data for each scan are available online at D.F. Levinson's website (accessed July 14, 2004) [8], and we use these data to reconstruct first the unweighted GSMA statistics *S*_*j*_, *j *= 1, 2, ..., 120, corresponding to the 120 bins spanning the entire genome, then their preferred weighted versions. Lewis [7] had recommended weights for each individual study proportional to the square root of the number of affected cases for that study. From Levinson's website [8], the individual weights *w*_*i*_, *i *= 1, 2, ..., 20, are 2.32, 1.77, 1.20, 1.17, 1.17, 1.16, 1.15, 1.08, 1.03, 1.01, 0.95, 0.88, 0.80, 0.80, 0.68, 0.67, 0.59, 0.54, 0.53, 0.51, greater than a four-fold range.

**Figure 1:**
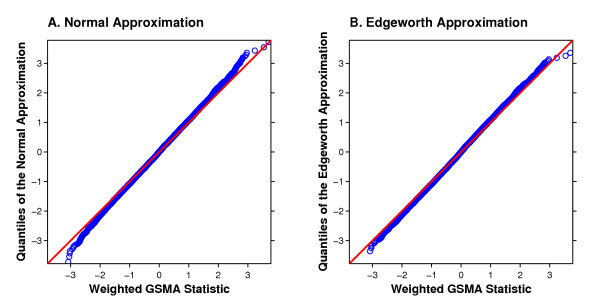
**Quantile-quantile plots of the weighted GSMA statistic vs. a normal approximation and an Edgeworth approximation**. **A**. Quantile-quantile plot of the null distribution of the weighted GSMA statistic, x-axis, versus the Edgeworth approximation, y-axis. **B**. Quantile-quantile plot of the empirical null distribution of the weighted GSMA statistic, x-axis, versus the Edgeworth approximation, y-axis. The empirical null distribution of the weighted GSMA statistic was obtained from 10,000 simulations, with *m *= 20 scans, *n *= 120 bins per scan, and weights for the 20 scans taken from Lewis [7]. The normal approximation shares the first two moments as the weighted GSMA statistic; the Edgeworth approximation shares the first four moments as the weighted GSMA statistic. Quantiles are depicted from the .0001 percentage point to the .9999 percentage point.

We simulated the null distribution of the weighted GSMA statistic 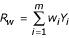, where 
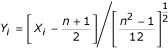
 with *m *= 20, *n *= 120, by drawing each *X*_*i *_as an independent random integer from 1 to 120 (that is, a uniform distribution of the integers from 1 to 120), then forming *R*_*w *_with the Levinson [8] weights. We used the random number generator in R [9], to produce 10,000 values for *R*_*w*_. We then formed 
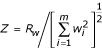
, and compared the empirical distribution of the 10,000 *Z *values with the Edgeworth approximation as described above; for comparative purposes, we also computed the normal approximation, which is based on matching the first two moments rather than the first four moments with Edgeworth. Figure 1 shows the resulting quantile-quantile plot of the empirical distribution of the weighted GSMA *Z *values with both a normal approximation, panel A, and the Edgeworth approximation, panel B. Note that, even in this setting of the weighted combination of m = 20 individual GSMA statistics, the normal approximation is particularly ill-fitting in the tails. Agreement in the tails would be of particular relevance in practical applications, as these represent the areas of potentially significant findings (p-values). The noticeable disagreement in the tails between the weighted GSMA statistic and its normal approximation is largely ameliorated with the Edgeworth approximation. With attendant computational savings, the Edgeworth approximation provides a practical means of determining significance of weighted GSMA results compared to simulation; tail probabilities derived from the normal approximation should only be used with extreme caution.

### The GSMA order statistics

We turn next to order statistic considerations (and reintroduce the subscript *j*). The Levinson [3] order statistic approach to inference relating to the GSMA statistics takes into account the inherent ordering of the *S*_*j*_: their *P*_*ord *_refers to the probability of any observed *S*_*k *_given the *k*^*th *^bin's place in the ordering of all of the *S*_*j*_. We here derive approximations to this distribution. Let 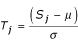, *j *= 1, ..., *n*, and *T*_(1) _<*T*_(2) _< ... *T*_(*n*) _denote the order statistics. We note first that the *T*_*j *_have (approximately) a singular symmetric multivariate normal distribution, with means 0, variances 1, and correlations 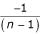. That the joint distribution is singular follows from the observation that 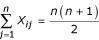 for all *i*, hence, 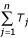 is identically 0. If we dismiss the correlations as negligible (of absolute magnitude < 0.01 for *n *> 100), then the *T*_*j *_are (approximately) independent, identically distributed *N*(0,1) (standard normal) random variates, and the cumulative distribution function (cdf) *F*_*k *_of the *k*^*th *^order statistic *T*_(*k*) _is given by


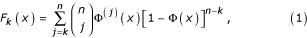


with Φ(·) as above (David [10], eqn. 2.1.3).

We briefly examine whether correlations can be ignored when determining the distributions of the *T*_(*j*)_. Numerical computation of the distributions of the order statistics from a symmetric multivariate normal distribution is feasible in a number of cases; we here examine perhaps the most relevant case, concerning the extreme *T*_(*n*). _Note that

**Figure 2:**
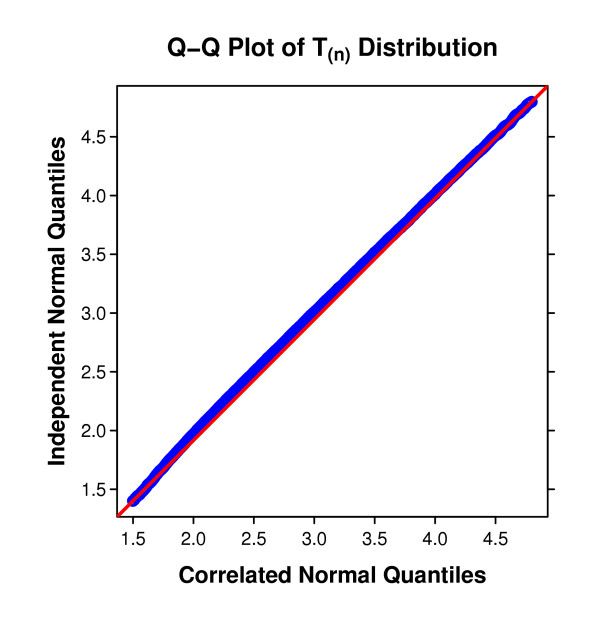
**Quantile-quantile Plot of *T*_(*n*) _Distribution**. Quantile-quantile plot of the approximate normal distribution of *T*_(*n*)_, the largest (order) GSMA statistic, under the correlation model (eqn. 2, with pairwise correlations 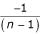), x-axis, versus the independence model (eqn.1), y-axis. Following Wise [1], we chose *n *= 120. Quantiles are depicted from the .001 percentage point to the .999 percentage point.

*Prob *(*T*_(*n*) _≤ *x*) = *Prob *(*T*_1 _≤ *x*,*T*_2 _≤ *x*, ...,*T*_*n *_≤ *x*);     (2)

this latter probability may be calculated in R using the mvtnorm package [9], based on methodology by Genz [11,12]. With *n *= 120, we depict in Figure 2 a Q-Q plot of the (approximate) distribution of *T*_(*n*) _under independence, eqn. (1), compared to the distribution from eqn. (2) with pairwise correlations 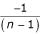. The independence model tends to agree quite closely to the correlation model in this particular case, especially in the critical right tail, and has the virtue of numerical simplicity. We remark that one might improve slightly on the normal independence model by incorporating the Edgeworth correction into the individual cumulative distribution functions in equation (1).

### Aggregate criteria and multiple testing

Levinson [3] had proposed an aggregate criterion for detecting linkage based on both the marginal distributions and the order statistic distributions of the GSMA statistics. In particular, they argued that bins that have achieved both *P*_*AvgRnk *_< 0.05 and *P*_*ord *_< 0.05 "are the most likely to contain linked loci or to be adjacent to linked bins". Note that their criterion entails both the marginal distribution of the *T*_*j*_, through *P*_*AvgRnk*_, and the (joint) order statistic distribution of the *T*_*j*_, through *P*_*ord*_. We remark that there is some redundancy to the aggregate criterion {*P*_*AvgRnk *_< 0.05 and *P*_*ord *_< 0.05}, as can be seen through consideration of critical values relating to their aggregate criterion. With the normal approximation to the distribution of each normalized GSMA statistic *T*_*j*_, the criterion {*P*_*AvgRnk *_< 0.05} is equivalent to the criterion {*T*_*j *_> 1.645}. The criterion {^*P*^*ord *< 0.05} may be computed from eqn. (1), and depends on the ordering of the individual *T*_*j*_. With *n *= 120, then for the ten largest order statistics *T*_(120)_, *T*_(119)_, ..., *T*_(111)_, the criterion {*P*_*AvgRnk *_< 0.05 and *P*_*ord *_< 0.05} reduces to {*P*_*ord *_< 0.05}, since their 95^th ^percentiles under their joint order statistic distribution exceed 1.645 [implying that, if {*P*_*ord *_< 0.05} obtains, then {^*P*^*AvgRnk *< 0.05} will automatically be satisfied]; and, for the remaining order statistics *T*_(110)_, *T*_(109)_, ..., *T*_(1)_, the criterion {*P*_*AvgRnk *_< 0.05 and *P*_*ord *_< 0.05} reduces to {*P*_*AvgRnk *_< 0.05}, equivalently, {*T*_(*j*) _> 1.645}, as their 95^th ^percentiles under the order distribution, eqn. (1), are less than 1.645 [implying that, if {*P*_*AvgRnk *_< 0.05} obtains, then {*P*_*ord *_< 0.05} will automatically be satisfied].

We conclude with a remark concerning multiple testing. Levinson [3] suggested a simple Bonferroni correction for multiple testing when determining the significance of GSMA results. In particular, they used the criterion {*P*_*AvgRnk *_< 0.000417} (0.05 corrected for 120 tests) for evidence that a bin is likely to contain a linked locus or loci. One can improve on this procedure by using Holm's [13] paradigm for multiple testing rather than Bonferroni. We illustrate Holm's [13] procedure by returning to the Lewis [7] study with *m *= 20 schizophrenia genome scans. As noted above, we used the online data to reconstruct the normalized unweighted GSMA statistics *T*_*j*_,*j *= 1, 2, ..., 120, corresponding to the 120 bins spanning the entire genome. With *m *= 20 studies, we shall invoke the normal approximation to the distributions of the individual *T*_*j*_.

Lewis [7] had extensively investigated various criteria for linkage from the 20 schizophrenia genome scans, and we shall not reproduce their analyses. Rather, we here illustrate a graphical procedure for the simultaneous evaluation of p-values from tests on the same data; this procedure is immediately applicable to the simultaneous consideration of the 120 GSMA statistics. The procedure, originally suggested by Schweder and Spjøtvoll [14], consists of a probability plot of the p-values versus the uniform distribution. Koziol [15] subsequently suggested that Holm's [13] paradigm for multiple testing be applied to Schweder and Spjøtvoll's [14] scenario, for a graphical determination of the number of true hypotheses.

Let us briefly review the Holm [13] method, which is an extension of the Bonferroni method for multiple comparisons. Suppose we compare the smallest p-value *P*_(1) _among *n *p-values with *α*/*n *and we find that the p-value is less than *α*/*n*. Then our multiple testing problem has been reduced by one test, and we should compare the next smallest p-value *P*_(2) _to 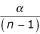. In general, we would compare *P*_(*i*) _with 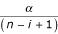. Holm's [13] step-down test begins with *i *= 1, comparing *P*_(*i*) _with 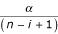, and stops as soon as *P*_(*i*) _exceeds 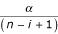, rejecting at overall level *α *all prior tests with smaller p-values. The Holm [13] method, like Bonferroni, makes no assumption on the dependence of tests, but beyond *P*_(1) _is less conservative than Bonferroni.

**Figure 3:**
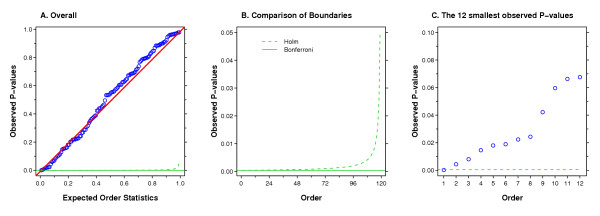
**Probability plot of GSMA schizophrenia statistics**. **A**. Probability plot of the 120 p-values corresponding to the 120 GSMA statistics *T*_1 _, ..., *T*_120 _from the 20 schizophrenia genome scans reported in Lewis [7], versus the (expected) uniform distribution. Also depicted are the Bonferroni (solid line) and Holm (dotted line) boundaries at overall alpha level 0.05. **B**. Inset of Figure 3A, in which we display solely the Bonferroni and Holm boundaries. We have rescaled the y-axis so as to emphasize the differences in the boundaries, and have relabeled the x-axis to correspond to the fact that we here have *n *= 120 ordered p-values. **C**. Inset of Figure 3A, illustrating the 12 smallest ordered p-values (circles), along with a Holm [13] boundary (solid line) at overall alpha level 0.05. [We are using the integer ordering of the x-axis as in Figure 3B.] Only the first p-value, corresponding to bin 2.5, falls outside this boundary. The bins depicted here, from left to right, are: 2.5, 3.2, 11.5, 5.5, 20.2, 8.2, 6.1, 2.6, 22.1, 1.6, 1.7, and 5.3.

In Figure 3A we present a probability plot of the 120 p-values corresponding to the 120 individual *T*_*j *_statistics, which we have recomputed from the online Levinson dataset [8]. On this plot, the points corresponding to the "true" hypotheses of no linkage in individual bins should roughly fall along a straight line passing through the origin. We have also superimposed the Bonferroni and Holm boundaries for overall alpha level 0.05 and n = 120 p-values; but, the two boundaries are virtually indistinguishable. There is little indication of large departures from the global null hypothesis of no linkage.

In Figure 3B we rescale the y-axis, and focus solely on the Bonferroni and Holm boundaries. Differences are most readily apparent for the largest ordered p-values. On the other hand, with a large number of hypotheses (here 120), the improvement of Holm over Bonferroni at the smallest ordered p-values is marginal at best. As a reviewer has presciently remarked, the Holm procedure generally is most helpful (advantageous) relative to Bonferroni with only a small number of hypotheses.

In Figure 3C we zoom in on the part of the probability plot nearest the origin; we here have superimposed the Holm [13] boundary. In accord with Lewis [7], we find that only one GSMA statistic achieves statistical significance at overall alpha level 0.05, namely, the statistic corresponding to bin 2.5. [Recall that the Holm and Bonferroni boundaries coincide at the smallest p-value, *P*_(1)_.] That is, in this particular instance, the unweighted GSMA statistics with either Bonferroni or Holm [13] correction for multiple testing identify statistically evidence for linkage on chromosme 2q.

## Conclusion

For practitioners utilizing GSMA statistics, the question arises as to whether the methods proposed here as well as in Koziol and Feng [2] are merely of theoretical interest, or have practical import. If one utilizes solely the unweighted GSMA statistic, and chooses to consider its marginal distribution (corresponding to the *P*_*AvgRnk *_formulation of Levinson [2]), then the exact null distribution of the GSMA statistic is available from Wise [1] or Koziol and Feng [2], and should be preferred over any approximate methods. If the exact null distribution is computationally intractable for practitioners, then the Edgeworth approximation of Koziol and Feng [2] provides a simple and accurate means of assessing significance; we would argue that the Edgeworth approximation is preferable to a normal approximation in this instance. When weights are introduced into the GSMA statistic, then the combinatoric arguments of Wise [1] and Koziol and Feng [2] will typically be insufficient to derive the exact null distribution [though we remark that a moment generating function approach patterned after the probability generating function formulation of Koziol and Feng [2] can be brought to bear on this problem.] One can either simulate the null distribution or derive an Edgeworth approximation: we do not believe either method enjoys global advantages over the other. We caution against simple reliance on a normal approximation: in the situation investigated here, Figure 1, the weighted combination of *m *= 20 individual GSMS statistics, the normal approximation is particularly ill-fitting in the tails. [Agreement in the tails is of particular relevance to practitioners, as these represent the areas of potentially significant findings (p-values).] As for the order statistic formulation and the aggregate criteria of Levinson [3], we believe that the theoretical considerations given in this paper can lead to clarification and simplification of testing criteria.

## Authors' contributions

JAK conceived, designed and drafted the manuscript. ACF performed the statistical simulation.

Both authors read and approved the final manuscript.
